# Ursodeoxycholic acid protects against lung injury induced by fat embolism syndrome

**DOI:** 10.1111/jcmm.15985

**Published:** 2020-11-04

**Authors:** Fangfang Niu, Huafei Li, Xiaotao Xu, Lingling Sun, Ning Gan, Aizhong Wang

**Affiliations:** ^1^ Department of Anesthesiology Shanghai Jiao Tong University Affiliated Sixth People’s Hospital Shanghai China; ^2^ Precision Medicine Center Taizhou Central Hospital (Taizhou University Hospital) Taizhou China; ^3^ School of Life Sciences Shanghai University Shanghai China

**Keywords:** acute lung injury, acute respiratory distress syndrome, fat embolism syndrome, ursodeoxycholic acid

## Abstract

Acute lung injury (ALI)/acute respiratory distress syndrome (ARDS) is a life‐threatening disease with a high mortality rate, which was a common complication of fat embolism syndrome (FES). Ursodeoxycholic acid (UDCA) has been reported to exert potent anti‐inflammatory effects under various conditions. *In vivo*, perinephric fat was injected via tail vein to establish a rat FES model, the anti‐inflammatory effects of UDCA on FES‐induced lung injury were investigated through histological examination, ELISA, qRT‐PCR, Western blot and immunofluorescence. *In vitro*, human lung microvascular endothelial cells (HPMECs) were employed to understand the protective effects of UDCA. The extent of ALI/ARDS was evaluated and validated by reduced PaO_2_/FiO_2_ ratios, increased lung wet/dry (W/D) ratios and impaired alveolar‐capillary barrier, up‐regulation of ALI‐related proteins in lung tissues (including myeloperoxidase [MPO], vascular cell adhesion molecule 1 [VCAM‐1], intercellular cell adhesion molecule‐1 [ICAM‐1]), elevated protein concentration and increased proinflammatory cytokines levels (TNF‐α and IL‐1β) in bronchoalveolar lavage fluid (BALF). Pre‐treatment with UDCA remarkably alleviated these pathologic and biochemical changes of FES‐induced ALI/ARDS; our data demonstrated that pre‐treatment with UDCA attenuated the pathologic and biochemical changes of FES‐induced ARDS, which provided a possible preventive therapy for lung injury caused by FES.

## 1 INTRODUCTION

Fat embolism syndrome (FES) is a type of embolism induced by fatty embolus in the circulation, which was caused by various factors including long bone fractures, orthopaedic trauma, bone marrow transplant and pancreatitis. Typical clinical features of FES are respiratory insufficiency and neurological dysfunction, as well as petechial rash, which start from 12 to 36 hours following major trauma. Owing to the physiological characteristics of the circulatory system, fat particles block the pulmonary capillaries and disrupt endothelial barrier, resulting in acute lung injury (ALI)/acute respiratory distress syndrome (ARDS).^1^ Besides, FES causes multiple injury in a variety of important organs, including liver, heart, brain and kidney.^2^ Currently, there are no definitive diagnostic criteria for FES, and the treatments are largely supportive and non‐specific. Therefore, the discovery and development for novel treatments become necessitated.

Ursodeoxycholic acid (UDCA), existing as a small amount of secondary cholic acid, is a 7β‐isomer of chenodesoxycholic acid, which is usually used for primary biliary cirrhosis (PBC).^3^ UDCA has been reported to have extensive protective actions and is recognized to be more secure among bile acids. Presently, no consensus has been reached on the mechanisms behind these beneficial effects of UDCA. In addition to promoting bile secretion, the immunomodulatory effects of UDCA have also been investigated.^4^, ^5^ Furthermore, UDCA was shown to own the effects for ameliorating airway inflammation via suppressing dendritic cell function.^6^ However, whether UDCA has protective effects on FES‐induced lung injury remains unknown.

In the current study, we intend to study the protective effects of UDCA in FES‐induced lung injury. For this purpose, we successfully established FES models in rats, based on which, the anti‐inflammatory effects were investigated. Our experimental results demonstrated that UDCA obviously attenuated FES‐induced lung inflammation both in rats and in HPMECs, which might be a useful candidate for lung inflammatory diseases.

## 2 MATERIALS AND METHODS

### 2.1 Reagents

UDCA, sodium‐oleic acid, sodium‐palmitic acid, stearic acid and arachidonic acid were purchased from Sigma‐Aldrich. TNF‐α and IL‐1β EILSA kit were from R&D Systems.

### 2.2 Animals

Animal experiments and procedures complied with the Institutional Animal Care and Use Committee of Shanghai Jiaotong University Affiliated Sixth People's Hospital, and conducted according to the Guide for the Care and Use of Laboratory Animals published by the National Institutes of Health. All possible efforts were made to minimize animal suffering and reduce the number of rats needed.

Adult male SD rats (8‐week) were assigned into 4 groups (n = 8) randomly, rats were intravenouly administrated with saline, perinephric fat (500 µL/kg, injection rate: 0.1 mL/min), saline + UDCA pre‐treatment, perinephric fat + UDCA pre‐treatment, respectively. The UDCA was only administrated 1 h before fat injection. At 12 h after all treatments, the rat lungs were harvested for further studies.

### 2.3 Perinephric fat preparation

Allogeneic rats were anesthetized with pentobarbital sodium (80 mg/kg, i.p.), the adipose tissues were isolated and the fat homogenate was prepared using an ultrasonic cell breaker, then the homogenate was centrifuged at 4°C (3000 *g* × 10 minutes). The whole process was carried out under pathogen‐free condition, and the supernatant was stored at −20°C for further studies.

### 2.4 Perinephric fat injection

The collected fat solution stored at −20°C was put into a electric‐heated thermostatic water bath to be rewarmed. Repeat centrifugation if the solution was not clear and transparent. Then, the rats were drived into a rat retainer to expose the tail (Yuyan Instruments). We sterilized the injection area and injected the fat via tail vein using a microsyringe. After injection, we applied pressure with sterile cotton balls to stop bleeding. Finally, we returned the rats to their cages and monitored their vital signs closely.

### 2.5 Oil red O staining

The lung tissues were collected after all treatments. After fixation and dehydration, the tissues were cut into slices about 8~10 µm thick. Fresh or frozen lung tissue sections were prepared with propylene glycol for 2 mins and then incubated using oil red O solution for 6 mins. Next, a mixture of 85% propylene glycol were applied for differentiation; then, the sections were rinsed for 2 times. Finally, sections were incubated in haematoxylin for 1‐2 mins and rinsed for 2 times.

### 2.6 Immunofluorescence

Confluent HPMEC monolayers grown on gelatin‐coated coverslips were subjected to immunofluorescent staining. After treatment, cells were fixed with 4% paraformaldehyde and permeabilized with 0.5% Triton X‐100. Rhodamine‐labelled phalloidin (Sigma) and VE‐cadherin antibody were used to visualize F‐actin filaments and adherens junctions. Cell nuclei were stained with DAPI (Beyotime Institute of Biotechnology, Haimen, China) at room temperature. After labelling, cells were rinsed to remove excessive label and examined using a confocal laser‐scanning microscope (Leica).

### 2.7 Quantitative real‐time reverse transcription PCR (qRT‐PCR)

Total RNA was extracted from lung tissues using trizol reagent (Invitrogen) following the manufacturer's instructions. Total RNA was reversely transcribed into cDNA, and then, equal amounts of the cDNA products were applied for PCR amplification using the qRT‐PCR thermal cycler (steponeplus, ABI). The expression values of MPO and P‐selectin were normalized with β‐actin mRNA levels.

### 2.8 Enzyme‐Linked ImmunoSorbent Assay (ELISA)

TNF‐α and IL‐1β levels were assayed by Quantikine ELISA kits according to the manufacturer's instructions.

### 2.9 Analysis of arterial blood gas

To analyse arterial blood gas, rats were randomly allocated into four groups (n = 8) and, respectively, injected with saline, UDCA (20 mg/kg), perinephric fat (500 µL/kg) and perinephric fat + UDCA. 12 h after administration, rats were anaesthetized and 2 mL arterial blood was drawn through the left carotid artery, blood samples were immediately analysed using a blood gas analyzer (Radiometer). Levels of PaO_2_ and PaO_2_/ FiO_2_ were all recorded.

### 2.10 BALF samples collectection

Rats were anesthetized with pentobarbital sodium (80 mg/kg, i.p.). BALF was obtained at 12 h after treatments by placing a catheter into the trachea, through which 1 mL of cold phosphate buffer saline (PBS) was flushed back for 3 times. Samples were determined for cell counting and centrifuged for 300 *g* × 10 minutes at 4°C with the supernatant being analysed for inflammatory cytokines. Protein concentration in the cell‐free BALF was determined using a BCA protein assay kit (Thermo Fisher, Scientific). The BALF neutrophils were calculated using Wright staining. TNF‐α and IL‐1β levels were assayed by Quantikine ELISA kits according to the manufacturer's instructions.

### 2.11 Survival analysis

Adult male SD rats were divided into four groups (n = 10) randomly and injected with saline, UDCA (20 mg/kg), perinephric fat (500 μL/kg) and perinephric fat + UDCA, respectively. Survival was monitored till 96 h. Efforts were made to minimize rat suffering and humane endpoints (weight loss ≥ 20% of pre‐experimental bodyweight) were used.

### 2.12 Pathological evaluation

We used separate sets of rats for pathological evaluation. Slices used for haematoxylin and eosin staining were prepared following standard methods. Severity of lung injury was evaluated according to the following criteria: interstitial inflammatory infiltration, oedema, haemorrhage, atelectasis and hyaline membrane. A semi‐quantitative scoring system was employed to evaluate the lung injury, and the scoring criterions were as follows: 0: normal appearance, 1: mild interstitial hyperaemia, polymorphonuclear leucocyte infiltration, 2: perivascular oedema and moderate pulmonary structural damage, 3: massive cell infiltration and moderate alveolar structure destruction, 4: massive cell infiltration and severe lung structural damage.^7^, ^8^

### 2.13 Western blot

Equal amount of protein samples were loaded and separated on 10% SDS‐PAGE, and the separated bands were then electroblotted onto PVDF membranes. The membranes were blocked and then probed with related primary antibodies at 4°C overnight; the membranes were visualized with HRP‐conjugated antibody. Protein signals were detected by Image Quant Ai600 (General Electric Co.) using an enhanced ECL substrate (Thermo Fisher, Scientific). The protein expression was quantified using ImageJ software (National Institutes of Health). The targeted proteins were normalized to β‐actin for statistical analysis.

### 2.14 Cell experiments

Human pulmonary microvascular endothelial cells (HPMECs) were from ScienCell Research. Cells were cultured in endothelial cell medium containing 5% foetal calf serum (Gibco) and 1% endothelial cell growth supplement, then incubated at 37°C, 5% CO_2_.

HPMECs were treated with free fatty acids (FFAs) according to our previous studies.^9^ FFAs are the main products of fat decomposition; they are also the direct cause of injury. Thus, a mixed solution consisted of 25% sodium‐oleic acid, 25% sodium‐palmitic acid, 25% stearic acid and 25% arachidonic acid, which were dissolved in 0.01 mmol/L NaOH solution, was used to treat HPMECs with a final total FFAs concentration of 0.5 mmol/L.

### 2.15 Statistical analysis

Data were presented as their mean ± SEM (n = 8) and were analysed with Graph‐Pad Prism 7.0 (Graph Pad Software). Statistical differences among groups were determined via Gaussian distribution prior to one‐way analysis of variance (ANOVA) followed by Tukey's post hoc test. In the case of F achieved *P* < .05, Tukey's post hoc tests were further applied for analysis. Non‐parametric tests were used if data were not normally variated, *P* < .05 presented a statistical significance.

## 3 RESULTS AND DISCUSSION

**Figure 1:**
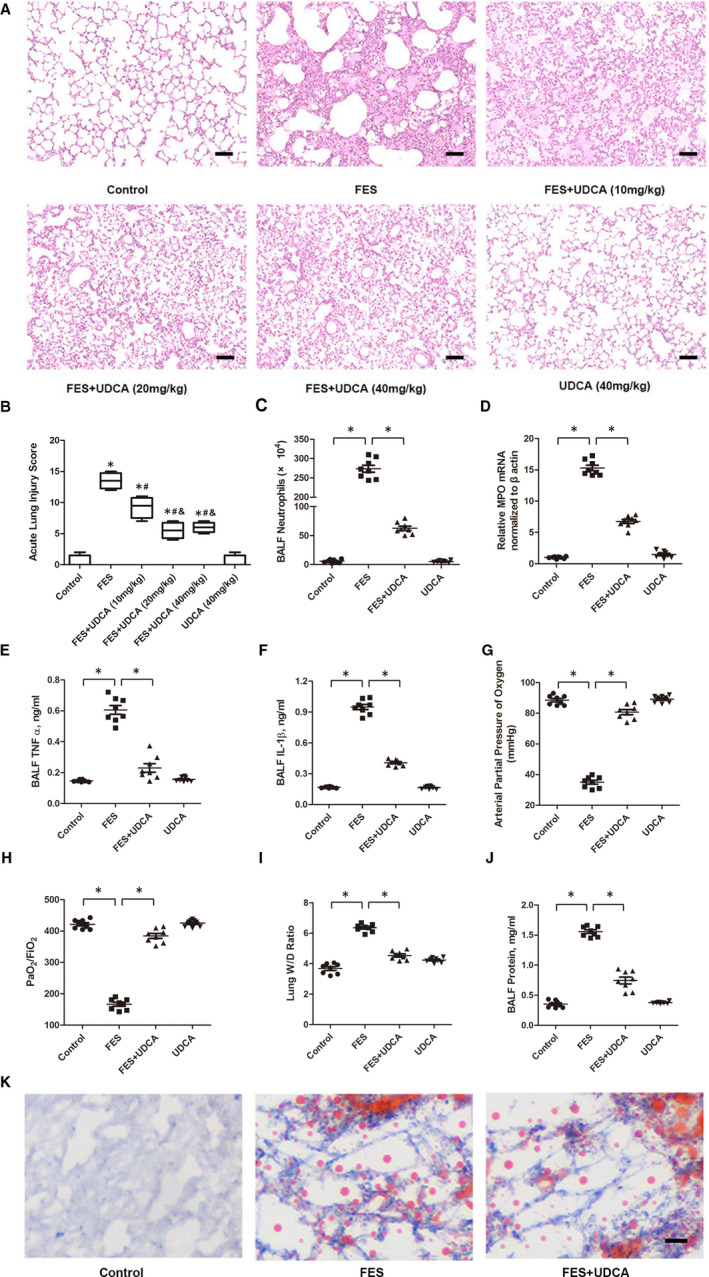
UDCA prevented FES‐associated lung injury. A, Representative H&E images from control, FES, FES plus 10 mg/kg UDCA, FES plus 20 mg/kg UDCA, FES plus 40 mg/kg UDCA and UDCA alone. B, Acute lung injury scores were calculated and statistically analysed among groups. (scale bar = 50 μm). C, BALF neutrophils, D, qRT‐PCR analysis of MPO expression, E, BALF TNF‐α concentration, F, BALF IL‐1β concentration, G, arterial pressure of oxygen analysis, H, PaO_2_/FiO_2_ ratios, I, Lung W/D ratios, J, Protein concentration of BALF. K, Oil red o staining of lung tissues at 30 mins after all treatments. (scale bar = 25 μm). The above parameters were determined at 12 h after treatments. Data are mean ± SEM, n = 8. *P < .05 vs the control group, ^#^ *P* < .05 vs the FES group. ^&^ *P* < .05 vs the FES + UDCA (10 mg/kg) group

Inappropriate accumulated neutrophils, dysregulated inflammation and altered alveolar‐capillary permeability are the major pathophysiological characteristics of ALI/ARDS. In this study, histopathological examination revealed that lung tissues of FES model rats suffered severe damages, being characterized by interstitial oedema, haemorrhage and inflammatory cells infiltration (Figure 1A,B). However, the results indicated that UDCA pre‐treatment but not post‐treatment improved pathological features of FES‐induced lung injury. This might due to faster occurrence and more severe symptoms of FES compared with LPS‐induced ALI/ARDS. It also raises the question whether the presence of UDCA prevented the delivery of fat emboli to the lung tissues. Oil red O staining was utilized to prove delivery of the fat emboli to target organs as opposed to a non‐specific inflammatory reaction leading to organ injury; the result showed that fat emboli dispersed evenly throughout the lung tissue in the FES + UDCA group similar to the FES group at 30 mins after all treatments (Figure 1K). This only implied that UDCA pre‐treatment did not affect the fat delivery to the lung at the beginning; then, lung tissue damage began to appear and become serious. At 12 h after all treatments, in the FES group, lung tissues showed severe impaired performances.

**Figure 2:**
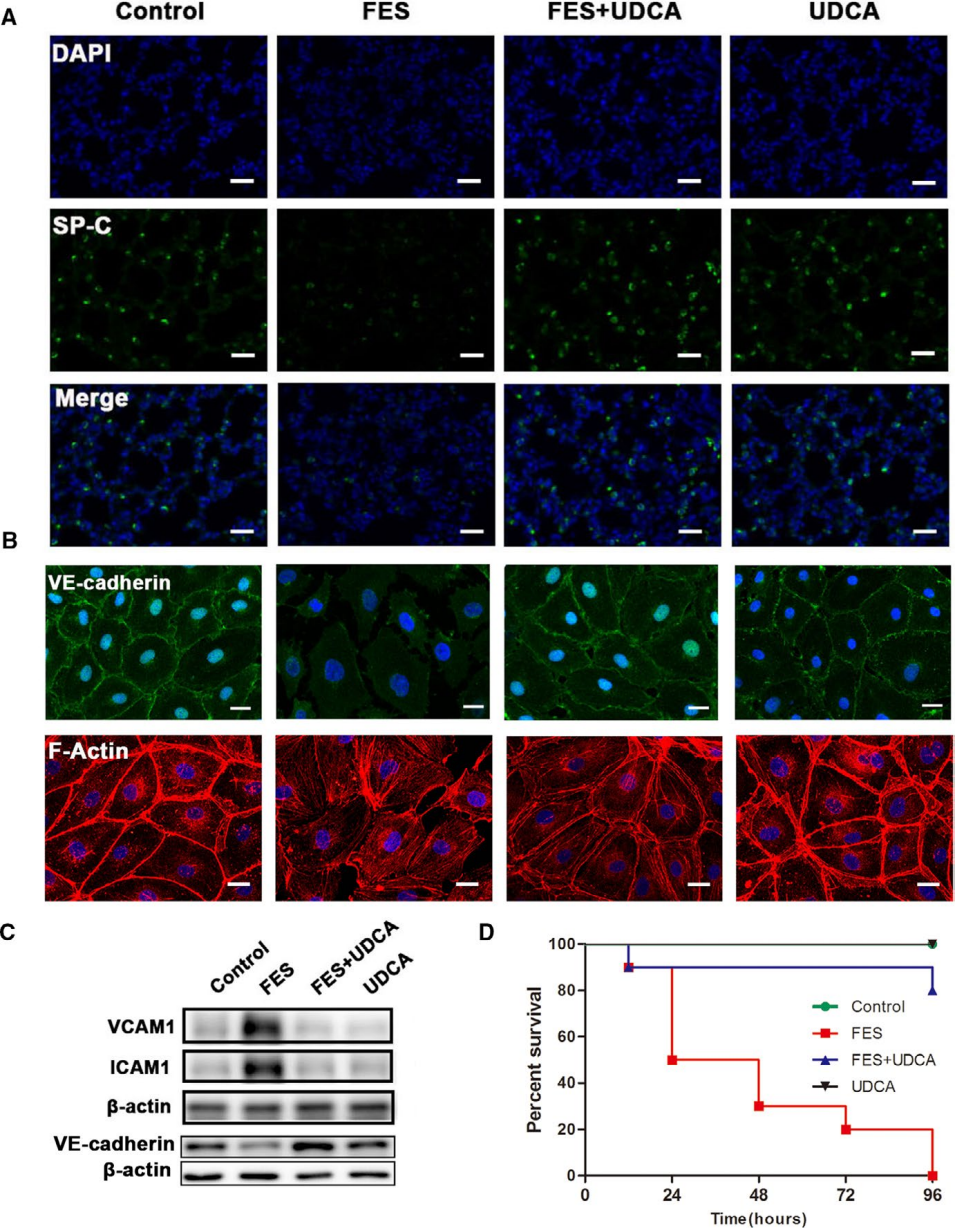
UDCA recovered ALI‐associated alveolar‐capillary dysfunction. A, Immunofluorescent staining of SP‐C in rat lung tissues. B, Confluent HPMEC monolayers grown on coverslips were treated with UDCA (1 mmol/L) for 1 h and then challenged with FFAs (0.5 mmol/L) for 12 h. Adherens junctions were examined by staining for VE‐cadherin (green); F‐actin was visualized by staining with phalloidin (red) (scale bar = 50 μm). C, Western blot analysis of VCAM1 and ICAM1 expression *in vivo* and VE‐cadherin expression in HPMECs. D, UDCA significantly prolonged the survival of FES model rats (n = 10 in each group). Data are mean ± SEM. n = 8. *P < .05

However, the UDCA treatment exerted obvious protective effects accompanied by reduced PaO_2_/FiO_2_, increased lung wet/dry (W/D) ratio and MPO activity, elevated proinflammatory factors (TNF‐α/IL‐1β) concentration, total protein and neutrophils in bronchoalveolar lavage fluid (BALF) in the FES + UDCA group (Figure 1C‐J). Consistent with our findings (Figure 1C), Mastrangelo et al's^10^ study revealed that neutrophil recruitment was involved in FFAs‐induced acute lung injury. As is well known, normal respiratory function depend on surfactant protein C (SP‐C) to reduce alveolar surface tension and prevent the alveolar collapse, FES‐induced down‐regulation of SP‐C was also largely reversed by UDCA (Figure 2A).

The following might be involved in the therapeutic effects of UDCA for alleviating FES‐associated lung injury. Firstly, inhibiting the production of inflammatory cytokines is of great significance to control pulmonary dysfunction. Inflammatory reactions enhanced due to the up‐regulation of inflammatory cytokines by stimulating the release of chemokines from epithelial cells and macrophages.^11^ In this study, UDCA reversed the TNF‐α/IL‐1β increase during FES‐induced lung injury. Secondly, intercellular junctions and cytoskeleton protein among endothelial cells are vital for endothelial integrity.^12^ UDCA administration significantly ameliorated the cytoskeleton protein (F‐actin) reorganization and the VE‐cadherin loss in HPMECs, which are of vital importance in the initiation and progression of FES‐induced lung injury (Figure 2B,C). Thirdly, the adhesion molecules VCAM1 and ICAM1 have been found to be involved in cell interactions during inflammatory responses.^13^, ^14^ In response to increased TNF‐α/IL‐1β, VCAM1 and ICAM1 were remarkably activated and up‐regulated in pulmonary microvascular endothelium (Figure 2C). Encouragingly, our experimental results indicated that UDCA pre‐treatment significantly attenuated all these pathological alterations and improved rat survival (Figure 2D).

Administration of UDCA largely reversed the reorganization of cytoskeleton protein (F‐actin) and the loss of VE‐cadherin in HPMECs, which are of vital importance in the initiation and progression of FES‐induced lung injury. FES‐induced oxidants generation were reported to activate the Rho family GTPases, including RhoA, Rac1 and cdc‐42.^15^, ^16^, ^17^ The small GTPases, including Rap1GTPases (Rap1a and Rap1b) and the Rho family GTPases (RhoA, Rac1 and Cdc42), played important roles in the homeostasis of endothelial actomyosin organization.^18^, ^19^, ^20^ By controlling the mechanical structure of endothelial cells through multiple pathways, these small GTPases regulated the endothelial barrier function.

Limitations also existed in this study. UDCA was proven to be prophylactically protective, but not therapeutically in FES‐associated lung injury. It is difficult to rule out the effects of UDCA on fat absorption, solubilization and metabolism. We are making efforts to clarify this question in our future work.

In summary, the current study showed that the administration of UDCA significantly attenuated ALI/ARDS in a rat FES model, which might offer a potential novel and effective precautionary measurement for lung injury.

## CONFLICTS OF INTEREST

The authors declare no conflict of interest.

## AUTHOR CONTRIBUTIONS

**Fangfang Niu:** Conceptualization (lead); Formal analysis (lead); Investigation (lead); Methodology (equal); Writing‐original draft (lead); Writing‐review & editing (lead). **Huafei Li:** Conceptualization (equal); Data curation (equal); Formal analysis (equal); Investigation (equal); Methodology (equal); Resources (equal); Software (lead); Validation (equal); Visualization (equal); Writing‐original draft (supporting); Writing‐review & editing (equal). **Xiaotao Xu:** Investigation (lead); Methodology (lead); Resources (lead). **Lingling Sun:** Formal analysis (equal); Investigation (supporting); Methodology (equal); Resources (equal); Software (equal); Validation (equal); Visualization (equal). **Ning Gan:** Data curation (equal); Methodology (equal); Resources (equal); Software (equal); Validation (equal); Visualization (equal). **Aizhong Wang:** Conceptualization (lead); Funding acquisition (lead); Investigation (lead); Project administration (lead); Resources (lead); Supervision (lead); Writing‐original draft (lead); Writing‐review & editing (lead).

## Data Availability

All data included in this study are available upon request by contact with the corresponding author.
